# The role of moderate‐vigorous physical activity and sedentary behavior on cognitive function: Cross‐sectional analysis in U.S. POINTER

**DOI:** 10.1002/alz.086670

**Published:** 2025-01-09

**Authors:** Shannon Halloway, Xiaoyan Leng, Pankaja Desai, Katie L Stone, Laura D Baker

**Affiliations:** ^1^ University of Illinois Chicago College of Nursing, Chicago, IL USA; ^2^ Wake Forest University School of Medicine, Winston‐Salem, NC USA; ^3^ Rush Institute for Healthy Aging, Chicago, IL USA; ^4^ Rush University Medical Center, Chicago, IL USA; ^5^ California Pacific Medical Center Research Institute, San Francisco, CA USA; ^6^ University of California, San Francisco, San Francisco, CA USA; ^7^ Wake Forest University, Winston‐Salem, NC USA

## Abstract

**Background:**

Healthy lifestyle behaviors can help prevent or delay cognitive impairment in older adults, including increasing moderate‐vigorous physical activity (MVPA). However, across studies examining effects of MVPA on cognitive function, findings are mixed. This may be partially explained by higher levels of sedentary behavior (SB) that may offset some benefit of MVPA. We aim to examine associations of MVPA and SB with cognitive function (global cognition, and the memory, executive function, and processing speed domains) and explore the degree to which SB offsets benefits of MVPA on cognitive function in older adults.

**Method:**

Cross‐sectional analyses were conducted using baseline data from 1680 participants from the U.S. POINTER trial, a lifestyle trial to prevent cognitive decline in at‐risk older adults without cognitive impairment at baseline. Cognitive function (global cognition and three domains) was comprised of composites of memory, executive function, and processing speed derived from pre‐specified scores of the test battery. MVPA and SB were assessed using validated self‐report questionnaires. Multiple linear regression was conducted to examine separate and combined effects of MVPA and SB on cognitive function. Additional models were conducted to examine the MVPA*SB interaction. Covariates included age, sex, racial and ethnic background, and education.

**Result:**

On average, participants were 68.4 years of age, 66.3% were of White racial background, 29.6% reported education less than college, and reported engaging in 4.3 hours/week of MVPA and 70.0 hours/week of SB. In the unadjusted models examining the separate effects of MVPA and SB with cognitive function, higher MVPA was related to better processing speed (*b* = .03, *p* = .005), while lower SB was related to better global cognition (*b* = ‐.002, *p* = .003), episodic memory (*b* = ‐.002, *p* = .026, executive function (*b* = ‐.002, *p*<.001), and processing speed (*b* = ‐.002, *p*<.001). These findings did not remain significant once adjusted for sociodemographics. The combined effects of MVPA and SB or the MVPA*SB interaction were not statistically significant.

**Conclusion:**

Although MVPA and SB were found to be each separately related to better cognitive function in older adults, associations did not remain once considering the influence of sociodemographics. In our upcoming analyses, we will examine associations using objective measures of MVPA and SB.

